# Lean DLY Pig-Derived Fecal Microbiota Promotes Growth Performance by Modulating Gut Microbiota: Serum Metabolic Profiles in Obese Ningxiang Pigs

**DOI:** 10.3390/ani16020177

**Published:** 2026-01-07

**Authors:** Li Han, Feng Zhou, Chen Zhang, Hongkun Li, Yongmin Zheng, Yv Tian, Yang Liu, Jie Yin, Xingguo Huang

**Affiliations:** 1 College of Animal Science and Technology, Hunan Agricultural University, Changsha 410128, China; hl68497075@126.com (L.H.); i_zhoufeng@stu.hunau.edu.cn (F.Z.); zhch@hunau.edu.cn (C.Z.); lhko328@163.com (H.L.); 19896222912@163.com (Y.Z.); tianyv121625@163.com (Y.T.); 2 Yuelushan Laboratory, Changsha 410128, China; 3 College of Biology and Food Engineering, Fuyang Normal University, Fuyang 236041, China; 4 Anhui Provincial Key Laboratory of Livestock and Poultry Product Safety, Institute of Animal Husbandry and Veterinary Medicine, Anhui Academy of Agricultural Sciences, Hefei 230011, China; 5 Hunan Institute of Animal Husbandry and Veterinary Medicine, Hunan Academy of Agricultural Science, Changsha 410015, China; yangl035@163.com

**Keywords:** fecal microbiota transplantation, Ningxiang pigs, gut microbiota, serum metabolism, growth performance, nutrient digestibility

## Abstract

Fecal microbiota transplantation (FMT) has demonstrated potential in reshaping gut microbiota to improve animal phenotypes, yet its application in lean-type to obese-type pigs like Ningxiang (NX) pigs remains unclear. To address this, we investigated the effects of Lean Duroc × Landrace × Yorkshire (DLY) pig-derived fecal microbiota on the growth, gut microbiota composition, and serum metabolism of obese NX pigs. Thirty-six 50-day-old castrated male NX pigs of similar initial body weight were randomly assigned to either a control group or FMT group. The trial lasted for 35 days. Results indicated that FMT significantly improved the average daily gain and increased nutrient digestibility. Serum biochemical analysis revealed elevated levels of globulin and total protein and reduced low-density lipoprotein cholesterol in the FMT group. In addition, 16S rRNA sequencing demonstrated that FMT modified gut microbiota composition and diversity, enriching beneficial genera such as *Blautia*, *Agathobacter*, *Faecalibacterium*, and *Eubacterium_coprostanoligenes*_group. Untargeted serum metabolomics further revealed altered metabolite profiles linked to lipid and amino acid metabolism. Correlation analysis further revealed a link between these enriched bacteria and metabolites changes. Overall, these findings demonstrate that transplantation of the fecal microbiota from lean DLY pigs significantly improved the growth performance of obese NX pigs by improving nutrient digestibility and modulating the gut microbiota–host metabolic axis.

## Simple Summary

The obese-type Ningxiang (NX) pig is renowned for its exceptional meat quality and flavor, yet its commercial potential is limited by lower feed efficiency and slower growth rates compared to lean-type commercial breeds. Fecal microbiota transplantation (FMT) has emerged as a promising approach to modulate host phenotypes via remodeling of the gut microbiota. Although previous studies have explored FMT from maternal or indigenous breed sources to lean-type pigs, the effect of transplanting fecal microbiota from lean-type pigs into obese-type breeds, particularly on growth performance and nutrient utilization, remains unclear. To address this, we hypothesized that transplanting fecal microbiota from lean-type Duroc × (Landrace × Yorkshire) (DLY) pigs into obese NX pigs would enhance growth performance and nutrient digestibility by modulating gut microbiota and host metabolism. In this study, we demonstrated that lean-type DLY-derived FMT significantly improved growth and nutrient digestibility in NX pigs, reshaped gut microbiota composition, and regulated serum metabolic profiles. These findings establish that lean pig-derived microbiota can effectively improve phenotypic traits in obese-type local breeds, offering valuable insights for optimizing swine production.

## 1. Introduction

Ningxiang (NX) pigs, an indigenous pig breed in China, are celebrated for their excellent flavor, superior meat quality, and environmental adaptability [1]. However, their commercial potential remains limited due to relatively low feed efficiency and slower growth rates compared to lean-type breeds. Therefore, improving the productivity of local breeds can play a crucial role in meeting the growing global demand for high-quality pork. Substantial evidence highlighted the significant differences in the composition of gut microbiota between lean and obese pigs. Lei et al. (2021) found that NX pigs showed a higher Firmicutes-to-Bacteroidetes ratio compared to Large White pigs [2]. Bama mini-pigs and Landrace pigs exhibit notable differences in colonic bacterial populations and metabolites [3], while distinct caecal metabolomic differences were observed between Jinhua and Landrace pigs [4]. Moreover, significant differences in serum metabolomic profiles existed between Min and Large White pig breeds [5]. These microbial differences could play an essential role in pig growth performance, nutrient utilization efficiency, and overall health.

Fecal microbiota transplantation (FMT) is as an emerging and promising technique that involves transferring a healthy donor’s entire commensal microbial community into the recipient’s gut, with the goal of rapidly reshaping the recipient’s microbiota [6]. This technique is gaining increasing attention from researchers. Several studies have confirmed the effectiveness of FMT in enhancing the diversity and composition of gut microbiota, as well as improving intestinal barrier function in recipient piglets [7,8]. For instance, transplantation of the fecal microbiota from Tibetan pigs to weaned Duroc × (Landrace × Yorkshire) (DLY) pigs [9], from Landrace sows to suckling piglets [10], and from Min sows to Yorkshire piglets [11] have all shown positive outcomes. Furthermore, FMT has been shown to enhance growth performance in recipient pigs [12], significantly enhancing the average daily weight gain (ADG) of recipient lean-type piglets receiving transplantation of the fecal microbiota from Landrace sows [10], adult Jinhua pigs [13], or NX pigs [14]. However, the outcomes of FMT application have not been entirely consistent across studies. For example, early transplantation of fecal microbiota from Rongchang pigs to DLY piglets reportedly disrupted the balance of gut microbiota and negatively affected intestinal barrier function [15], and FMT from DLY pigs had no significant impact on the ADG of the pigs’ offspring [9]. These discrepancies may be attributed to the source and health status of the donor (e.g., breed, age, phenotype), and the existing microbial community structure of the recipient prior to intervention. While much of the research has focused on transplantation of the fecal microbiota from maternal sources or indigenous breed pigs to lean-type pigs, the modulatory effects of transplantation of the fecal microbiota from lean-type donor pigs on the growth performance and gut microbiota composition of obese indigenous breeds remain unclear. Given the potential implications for improving the commercial viability of indigenous breeds, there is a pressing need to explore the effects of transplantation of the fecal microbiota from lean-type pigs on obese breeds, specifically with respect to growth performance and nutrient utilization. Our previous study indicated that gut microbiota profile differed between lean DLY pigs and obese NX pigs [16]. DLY pigs, as a lean-type model, whose microbiota transplantation might remodel host metabolism. We hypothesize that FMT from lean-type DLY pigs enhances the growth performance and nutrient digestibility of obese-type NX pigs by modulating gut microbiota composition and host metabolism. To preliminarily test this hypothesis, the study will focus on assessing the effects of FMT on the growth performance (including average daily gain and feed conversion ratio (FCR)) and nutrient digestibility of NX pigs. We characterize the changes in gut microbiota composition in recipient NX pigs following FMT using 16S rRNA gene sequencing and analyze alterations in serum metabolite profiles using untargeted metabolomics. This study aims to provide valuable insights into the potential applications of FMT for enhancing the commercial viability of indigenous pig breeds like the NX pig, and addressing the growing demand for high-quality pork.

## 2. Materials and Methods

### 2.1. Experimental Design and Diets

A total of 36 castrated male NX pigs at 50 d age with similar initial body weight (BW) (19.70 ± 0.5 kg) and a uniform genetic background were randomly allocated to two experimental groups: control group (fed a basal diet + 10 mL/day normal saline) and FMT group (fed a basal diet + 10 mL/day of fecal microbiota suspension from age-matched DLY pig). Six DLY pigs of similar age and weight to the NX pigs served as FMT donors and were housed in an environmentally controlled room. All donors were maintained on the same basal diet (NRC, 2012) [16] and clean water as the recipients (Table S1) to standardize the source microbiota. The trial duration was 35 days, with the first 7 days allocated for adaptation. Following this adaptation period, the fecal bacterial suspension from the DLY pigs was prepared daily. The preparation ratio, preparation methodology, preparation condition, and dosage of the fecal microbiota suspension primarily referenced studies by Hu, Yin, and Yang et al. [13,17,18]. In practice, DLY pig’s fecal microbiota suspension involved the collection of fresh fecal samples from the donor DLY pigs daily, at 8:00 a.m. Approximately 10 g of feces was collected from each pig and mixed. The pooled sample was then divided equally into six 50 mL centrifuge tubes (10 g per tube), then homogenized with 40 mL precooling normal saline, thoroughly mixed using a vortex shaker, and centrifuged at 3000 r/min for 10 min at 4 °C [17]. The resulting microbial supernatant was filtered three times through sterile gauze to remove large particles [19], yielding the fecal microbiota suspension. The resulting microbial supernatant could be mixed with diet for direct feeding [18]. The FMT group was supplemented with 10 mL of the freshly prepared DLY pig’s fecal bacterial suspension into the basal diet by 8:30 a.m., whereas the control group received an equal volume of sterile saline mixed following the same schedule. This procedure was carried out daily for both groups throughout the trial [16]. Environmental conditions were maintained at approximately 22–24 °C with 60–65% relative humidity, and all pigs had ad libitum access to both feed and water.

### 2.2. Growth Performance

On days 7, 32, and 35 of the experiment period, the BW of the NX pigs was measured after a 12 h overnight fast to calculate ADG. Daily feed intake for each pig was tracked by calculating the average daily feed intake (ADFI) and FCR.

### 2.3. Fecal Digestibility Coefficients of Nutrients

Fecal samples were collected from a randomly selected subset of ten pigs per group on day 32 of the experiment [20]. Fresh fecal samples (200 g/d) were collected during three consecutive days (days 32–34) through spontaneous defecation from ten pigs in control and FMT groups (0.25% titanium dioxide as an indigestible marker). The samples were placed in sterile cryopreservation tubes, and temporarily stored at −20 °C. Subsequently, the daily samples were composited in equal proportions. Fecal samples (300 g) were dried in an oven at 65 °C for 48 h using a forced-air drying oven (Model 101-19B Shinbae Industrial Co., Ltd., Shanghai, China), then sieved through a 0.425 mm screen via a centrifugal mill (Foss Tecator; Akutalstuku, Tokyo, Japan), and subsequently stored at −20 °C until subsequent nutrient digestibility coefficient analysis. The nutrient digestibility coefficients of dry matter (DM; method 927.05), ether extract (EE; method 2003.06), crude protein (CP; method 990.03), crude fiber (CF; method 991.43), calcium (Ca; method 985.01), and phosphorus (P; method 985.01) was analyzed and calculated according to AOAC Int., 2007 and 2019 standard methods [21].

### 2.4. Sample Collection and Serum Biochemical Indicators

The anterior vena cava blood was collected from a randomly selected subset of the ten pigs per group into vacuum anticoagulant tubes (Becton Dickson, Shanghai, China) at 8:00 a.m. (prior to the morning feeding) on days 35, and centrifuged at 3000 rpm (4 °C, 15 min) after stand for 30 min to obtain serum. The serum was transferred into a new sterile cryopreservation tube, then stored at −80 °C until the analysis of serum biochemical indicators and untargeted metabolomics [22,23].

The levels of low-density lipoprotein cholesterol (LDL-C), high-density lipoprotein cholesterol (HDL-C), triglyceride (TG), total cholesterol (TC), serum creatinine (CRE), globulin (GLB), albumin (ALB), albumin–globulin ratio (A/G), total protein (TP), total bile acid (TBA), and alanine aminotransferase (ALT) were measured using commercial reagent kits provided by Shanghai Kehua Bio-engineering Co., Ltd. (Shanghai, China), following the manufacturer’s instructions.

### 2.5. Sample Collection, Fecal Microbiota 16S rRNA Gene Sequencing, and Analysis

On day 35, fresh fecal samples were collected into the sterile tube during spontaneous defecation from a randomly selected subset of ten pigs per group, and stored at −80 °C for subsequent 16S rRNA gene sequencing. Genomic DNA was extracted following the instructions of the DNA extraction kit (Accurate Biology, Changsha, China) [24,25]. DNA integrity was assessed by 1% agarose gel electrophoresis, and its concentration and purity were measured with a NanoDrop2000 spectrophotometer (Thermo Scientific, Waltham, MA, USA) [26]. The extracted DNA was used as a template for PCR amplification of the V3-V4 regions of the 16S rRNA gene with primers 338F (5′-ACTCCTACGGAGGCAGCAG-3′) and 806R (5′-GACTACHVGGGTWTCTAAT-3′) [16,26]. PCR was performed with TransStart Fastpfu DNA Polymerase (TransGen BioTech, AP221-02, Beijing, China) in a 20 μL reaction volume. Subsequently, the AxyPrep DNA Gel Extraction Kit (Axygen Biosciences, Union City, CA, USA) was used to purify the product and verified for purity and quantity by gel electrophoresis and with a Quantus™ Fluorometer (Promega, Madison, WI, USA). The purified products were used to construct a library and sequenced on an Illumina Miseq platform (Illumina, San Diego, CA, USA) at Shanghai Majorbio Bio-pharm Technology Co., Ltd. (Shanghai, China).

Sequence data were processed with Fastp (v0.19.6) for quality filtering, denoising, and chimera removal. High-quality sequences were merged using FLASH (v1.2.11). Operational taxonomic units (OTUs) were clustered at a 97% similarity threshold using UPARSE (v7.1) and taxonomically annotated against the Silva 16S rRNA database (v138). Microbial diversity and community structure were assessed on the Majorbio platform (https://cloud.majorbio.com/) (accessed on 26 April 2025). Alpha diversity indices (Sobs, Ace, Chao1, Shannon, Simpson, and Good’s coverage) and rarefaction curves were calculated from OTU data using Mothur v1.30.1. Beta diversity, based on Bray–Curtis dissimilarity, was analyzed by PCoA visualization and similarity (ANOSIM) statistics testing. OTU analysis was performed to generate Venn diagrams representing unique and shared taxa across two groups. Community bar plots were constructed to visualize microbial composition at the phylum and genus levels. The linear discriminant analysis effect size (LEfSe) was analyzed to compare and identify significant different taxa microbes in abundance among groups, with FDR-adjusted *p* < 0.05 considered statistically significant (LDA score > 3, *p* < 0.05) [16,17].

### 2.6. Analysis of Serum Metabolomics

Serum metabolomics analysis was conducted using LC-MS. In brief, 100 μL of serum was mixed with 400 μL of extraction solution (200 μL acetonitrile and 200 μL methanol). The mixture was ultrasonically extracted for 30 min at 4 °C, incubated for 30 min at −20 °C, and then centrifuged at 13,000 rpm (4 °C, 15 min). The supernatant was discarded, and the residual solvent was evaporated under nitrogen gas. Subsequently, the residue was resuspended with a resuspension solution (60 µL acetonitrile and 60 µL water), ultrasonically extracted for 5 min at 5 °C, and centrifuged at 13,000 rpm (4 °C, 5 min) [14]. The resulting supernatant was collected into a new centrifuge tube for subsequent sequencing.

Analytical repeatability was monitored by analyzing pooled quality control (QC) samples (generated from equal aliquots of all serum specimens) following every fifth sample in the run. Untargeted metabolomic sequencing analysis was performed in both negative and positive ion modes by using a UPLC-TripleTOF system (AB SCIEX, Framingham, MA, USA). Raw data were processed with Progenesis QI software (v2.3, Waters Corporation, Milford, MA, USA) for data alignment, normalization, and peak picking, resulting in a final data matrix for subsequent analysis. Metabolites were annotated using Metlin (https://metlin.scripps.edu/) (accessed on 26 April 2025) and HMDB (https://www.hmdb.ca/) (accessed on 26 April 2025) databases. The preprocessed data was uploaded to Majorbio Cloud Platform (https://cloud.majorbio.com/) (accessed on 26 April 2025) to obtain the analysis matrix. The R package ropls (Version 1.6.2) performed partial least squares discriminant analysis (PLS-DA) and orthogonal partial least squares discriminant analysis (OPLS-DA), with ANOSIM testing intergroup differences. Key metabolites were identified according to the VIP > 1 (from OPLS-DA) and q-value < 0.05 (false discovery rate correction) of Student’s *t* test, then analyzed for metabolic pathway enrichment using the KEGG database (https://www.genome.jp/kegg/) (accessed on 26 April 2025) [14].

### 2.7. Statistical Analysis

Statistical analyses were conducted using IBM SPSS Statistics 22.0 (IBM Corporation, Armonk, NY, USA). Microbial differential analysis (based on relative abundance) was performed with the Kruskal–Wallis rank-sum test. Spearman’s correlation analysis was used to evaluate the relationships between gut microbiota, differential serum metabolites, and nutrient digestibility. Data were presented as the mean ± SEM, with *p* ≤ 0.05 considered statistically significant, *p* ≤ 0.01 regarded as a highly significant difference, and 0.1 > *p* > 0.05 considered indicative of a trend.

## 3. Results

### 3.1. Effects of FMT on Growth in NX Obese Pigs

To assess the impact of FMT on the growth of NX pigs, the ADG and ADFI were calculated through systematic measurement. As shown in Table 1, lean DLY pig-derived fecal microbiota had a significantly increased the ADG (*p* = 0.014) and reduced the FCR (*p* = 0.016) in obese NX pigs.

### 3.2. Effects of FMT on the Nutrient Digestibility in Obese NX Pigs

To investigate the impact of FMT on the nutrient digestibility in NX pigs, we evaluated the apparent digestibility of DM, EE, CP, CF, Ca, and P in the fecal samples. As presented in Table 2, lean DLY pig-derived fecal microbiota significantly increased the nutrient digestibility of Ca (*p* < 0.01), P (*p* < 0.01), and CF (*p* = 0.026) in obese NX pigs, and there was an improving trend observed in the nutrient digestibility of CP (*p* = 0.053) and ether EE (*p* = 0.083) in obese NX pigs.

### 3.3. Effects of FMT on Serum Biochemical Indicators in Obese NX Pigs

We evaluated the impact of the FMT on the serum biochemical indicators. As shown in Table 3, lean DLY pig-derived fecal microbiota significantly reduced the LDL-C level (*p* = 0.049), and significantly increased the levels of GLB (*p* = 0.027) and TP (*p* < 0.01) in obese NX pigs.

### 3.4. Effects of FMT on the Gut Microbiome in Obese NX Pigs

**Figure 1:**
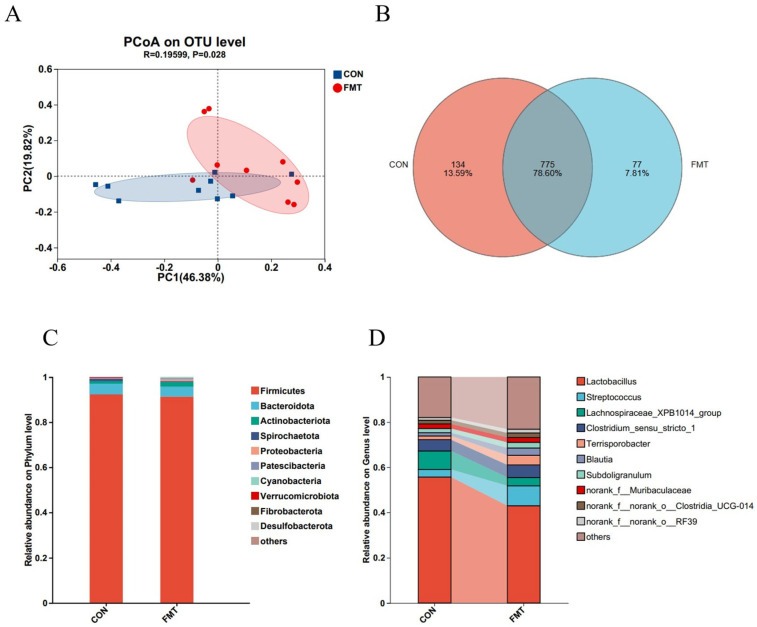
Effects of transplantation of the fecal microbiota from DLY pigs on the fecal microbiota diversity in NX pigs. Comparison of beta diversity (**A**), common OTU analysis (**B**), and the microbiota composition phylum (**C**) and genus (**D**) levels between CON and FMT group (top 10).

**Figure 2:**
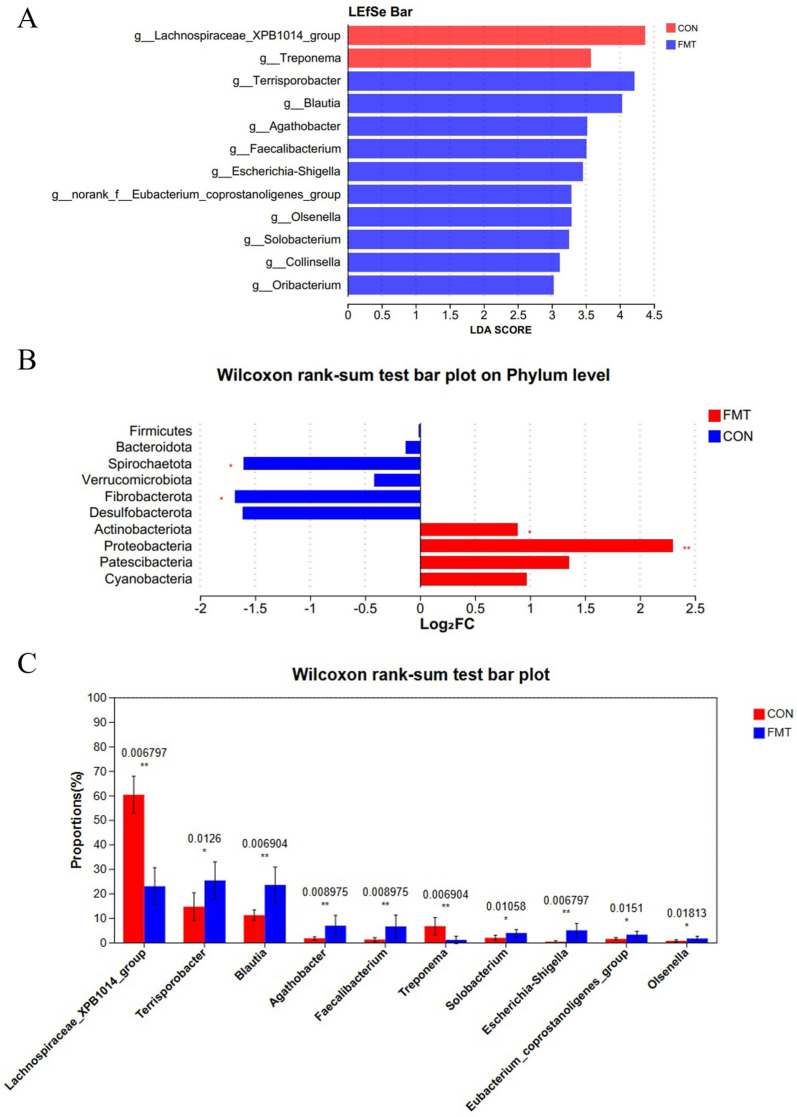
Effects of transplantation of the fecal microbiota from DLY pigs on the fecal microbial abundance in NX pigs. The enriched microbiota from kingdom level to genus level (LDA Score > 3) (**A**). Comparison of microbial abundance at the phylum and genus levels, displaying the top 6 phyla (**B**) and top 10 genera (**C**), respectively, ranked by sum of mean abundance values. *, 0.01 < *p* ≤ 0.05; **, *p* ≤ 0.01.

Next, 16S rRNA gene sequencing of the V3–V4 region retained a total of 914,703 sequences from all samples after QC. After subsampling to the minimum per-sample sequence depth, an average of 37,234 reads per sample were generated for subsequent species annotation and abundance analysis. Alpha diversity of the fecal microbiota differed significantly between two groups for the Chao1 and Ace indices (*p* < 0.05), but not for the Sobs, Simpson, Shannon indices, and Coverage (*p* > 0.05; Table 4). The sequencing depth was sufficient, confirming the reliability of the data. Beta diversity analysis showed significant differences (*p* < 0.05), with post-FMT samples displaying a more dispersed distribution based on PCoA distance (Figure 1A). Venn diagram analysis identified 775 shared OTUs between the control and FMT groups, with 77 unique OTUs in the FMT group (Figure 1B). At the phylum level, Firmicutes and Bacteroidetes dominated both groups, accounting for over 95.12% of total sequences (Figure 1C), with no significant differences in their relative abundances (Figure 2B) (*p* > 0.05). At the genus level, the control group was predominantly composed of *Lactobacillus*, *Lachnospiraceae*_XPB1014_group, *Clostridium*, and *Streptococcus*, while the FMT group exhibited the most abundance of *Lactobacillus*, *Streptococcus*, *Subdollgranulum*, *Clostridium*_sensu_stricto_1, *Terrisporobacter*, and *Blautia* (Figure 1D). LEfSe analysis (LDA score > 3) identified microbial biomarkers, with two in the control group and ten in the FMT group (Figure 2A). Notably, the relative abundance of *Lachnospiraceae*_XPB1014_group (*p* = 0.007) and *Treponema* (*p* = 0.007) was significantly higher in the control group (Figure 2C). Conversely, the relative abundance of *Blautia* (*p* = 0.007), *Agathobacter* (*p* = 0.009), *Eubacterium_coprostanoligenes*_group (*p* = 0.02), *Olsenella* (*p* = 0.02), *Terrisporobacter* (*p* = 0.01) and *Faecalibacterium* (*p* = 0.009) were enriched in the FMT group (Figure 2C). These findings were consistent with the LEfSe analysis results. A limitation to note is the absence of negative extraction controls in the microbiome workflow, which precludes a formal assessment of potential low-level contamination.

### 3.5. Effects of FMT on the Serum Metabolites in Obese NX Pigs

**Figure 3:**
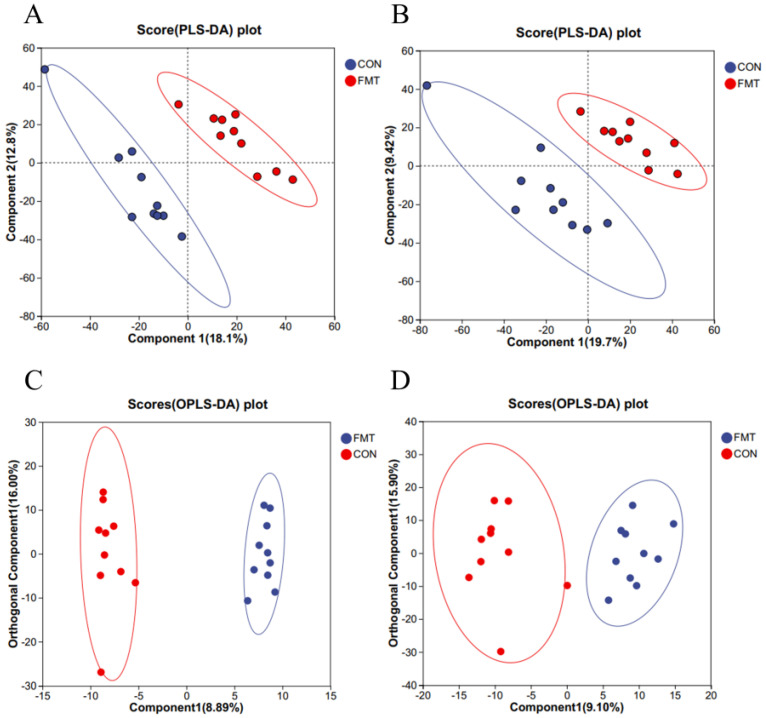
Effects of transplantation of the fecal microbiota from DLY pigs on the serum metabolic profiles of NX pigs. Partial Least Squares Discriminant Analysis (PLS-DA) plots (**A**,**B**); OPLS-DA plots (**C**,**D**) of serum metabolites in positive and negative ion mode.

**Figure 4:**
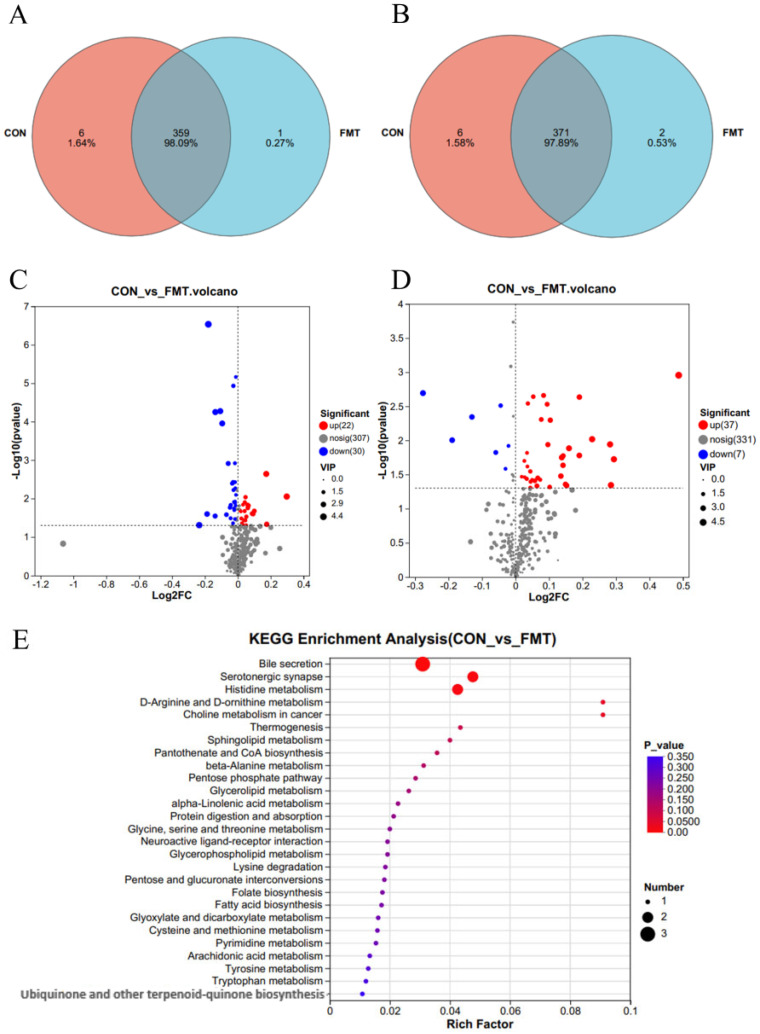
Effects of transplantation of the fecal microbiota from DLY pigs on the serum metabolic profiles of NX pigs. Venn diagram in positive (**A**) and negative (**B**) ion mode. Volcano plots in positive (**C**) and negative (**D**) ion mode. (**E**) KEGG pathway enrichment analysis.

To investigate the effects of lean DLY pig-derived microbiota on the metabolites in obese NX pigs, we conducted untargeted metabolomics using LC-MS to identify differential metabolites of obese NX pigs after FMT. PLS-DA (Figure 3A,B) and OPLS-DA (Figure 3C,D) revealed a distinct separation of metabolomes between the FMT and control groups. Venn diagram analysis identified 359 shared metabolites in cationic mode (Figure 4A). and 371 shared metabolites in anionic mode (Figure 4B). Differential metabolites were identified using a significance threshold of q < 0.05 and a VIP value > 1.0, based on the OPLS-DA model. A total of 96 metabolites showed significant alterations between the two groups after false discovery rate correction. Among these, 59 metabolites increased and 37 decreased after FMT (Figure 4C,D).

**Figure 5:**
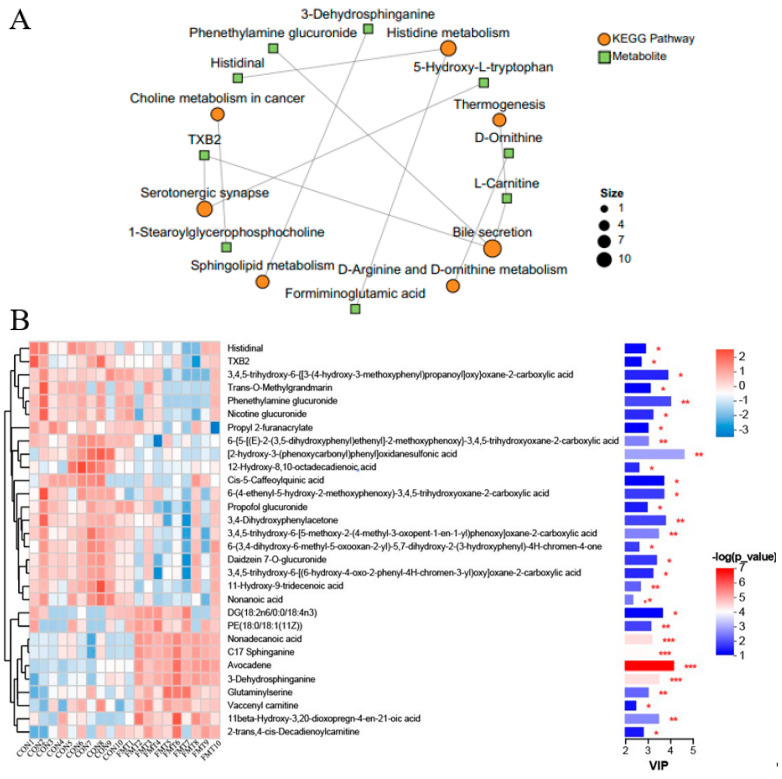
Effects of transplantation of the fecal microbiota from DLY pigs on the differential metabolites of NX pigs. (**A**) KEGG enrichment analysis network diagram of the top 10 differential metabolites (*p* ≤ 0.10). (**B**) The VIP scores and relative abundance of the top 30 differential metabolites. (* 0.01 < *p* ≤ 0.05, ** 0.001 < *p*≤ 0.01, *** *p* ≤ 0.001).

KEGG pathway analysis (top 30 ranked by *p*-value) indicated that differential metabolites mainly participated in several key metabolic pathways (Figure 4E), such as bile secretion (L-Carnitine, phenethylamine glucuronide), serotonergic synapse (TXB2, thromboxane B2), histidine metabolism (formiminoglutamic acid, histidinal), D-Arginine and D-ornithine metabolism (D-Ornithine), sphingolipid metabolism (3-Dehydrosphinganine), choline metabolism (1-stearoylglycerophosphocholine), tryptophan metabolism (5-hydroxy-L-tryptophan), with tyrosine metabolism (tyramine) constituting the core metabolic signature altered by FMT (Figure 5A). VIP analysis (top 30 ranked by VIP score) revealed that a number of metabolites showed significant changes between groups (Figure 5B). Importantly, the levels of 5-hydroxy-L-tryptophan (*p* = 0.03), tyramine (*p* = 0.037), aminoadipic acid (*p* = 0.022), formiminoglutamic acid (*p* = 0.039), histidinal (*p* = 0.033), N-formylmethionine (*p* = 0.028), phenethylamine glucuronide (*p* = 0.009), 1-stearoylglycerophosphocholine (*p* = 0.044), thromboxane B2 (TXB2) (*p* = 0.044), and L-carnitine (*p* = 0.013) were significantly decreased following FMT, whereas the levels of 3-dehydrosphinganine (*p* < 0.001), (2′E,4′Z,7′Z,8E)-colnelenic acid (*p* = 0.008), dodecanoic acid (*p* = 0.033), and glyceric acid (*p* = 0.012) were significantly improved following FMT.

### 3.6. Correlation Analysis

**Figure 6:**
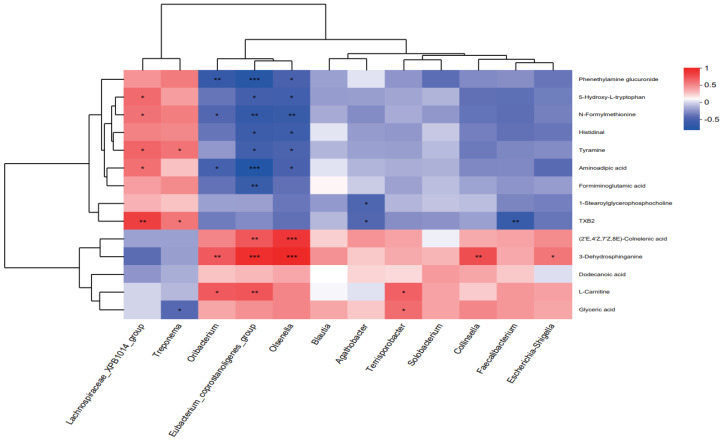
Correlation heatmap between differential microbiota and key metabolites. (* *p* ≤ 0.05, ** *p* ≤ 0.01, *** *p* ≤ 0.001).

**Figure 7:**
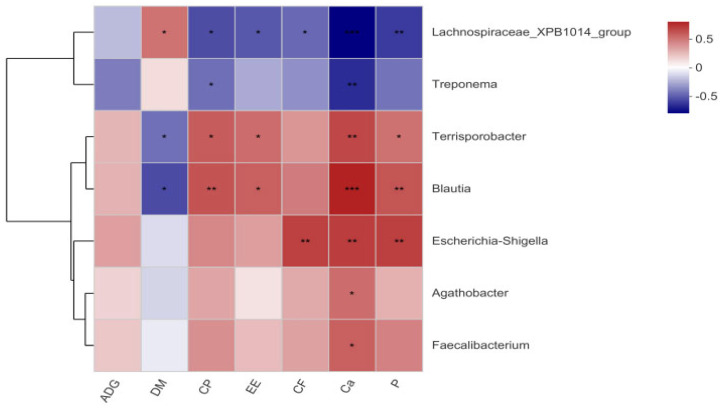
Correlation heatmap between differential fecal microbiota and ADG, indicating apparent digestibility. (* *p* ≤ 0.05, ** *p* ≤ 0.01, *** *p* ≤ 0.001).

Spearman’s correlation analysis was conducted to link differential microbiota (at the genus level, LDA > 3), metabolites, and nutrient digestibility (Figure 6 and Figure 7). The relative abundances of *Eubacterium_coprostanoligenes*_group (*p* < 0.001), *Oribacterium* (*p* = 0.010), *Olsenella* (*p* < 0.001), *Collinsella* (*p* = 0.007), and *Escherichia-Shigella* (*p* = 0.049) were positively correlated with the levels of 3-dehydrosphinganine. The relative abundance of *Lachnospiraceae*_XPB1014_group showed positive correlations with the levels of tyramine (*p* = 0.016), aminoadipic acid (*p* = 0.036), 5-hydroxy-L-tryptophan (*p* = 0.024) and N-formylmethionine (*p* = 0.035). Notably, the relative abundance of *Terrisporobacter* was positively associated with glyceric acid levels (*p* = 0.029), whereas the abundance of *Treponema* was negatively correlated with glyceric acid levels (*p* = 0.049). Furthermore, the relative abundances of *Oribacterium* (*p* = 0.021), *Eubacterium_coprostanoligenes*_group (*p* < 0.001), and *Olsenella* (*p* = 0.032) were negatively correlated with aminoadipic acid levels. Additionally, the levels of 5-hydroxy-L-tryptophan, histidinal, and tyramine also showed negative correlations with *Eubacterium coprostanoligenes* group (*p* = 0.011, *p* = 0.013, *p* = 0.022, respectively) and *Olsenella* (*p* = 0.018, *p* = 0.016, *p* = 0.035, respectively), while *Eubacterium coprostanoligenes* group was also negatively correlated with formiminoglutamic acid (*p* = 0.011) levels (Figure 6). In addition, we analyzed the associations between different microbiota and phenotypic indicators (Figure 7). Spearman’s analysis revealed that the nutrient digestibility of CP, EE, P, and Ca were positively correlated with *Terrisporobacter* (*p* = 0.009, *p* = 0.040, *p* = 0.052, *p* = 0.021, respectively) and *Blautia* (*p* = 0.016, *p* = 0.003, *p* = 0.012, *p* = 0.001, respectively). In contrast, the relative abundance of *Lachnospiraceae*_XPB1014_group was negatively correlated with the nutrient digestibility of CP (*p* = 0.058), P (*p* = 0.047), and Ca (*p* < 0.001).

## 4. Discussion

FMT in pigs involves transferring the entire commensal microbes from a healthy donor into the gut of a recipient pig, thereby reshaping the structure of the gut microbiota [8,27]. This strategy is gaining attention as a potential means to improve phenotypic traits. Previous research showed that early transplantation of the fecal microbiota from maternal sources or indigenous pig breeds to lean-type piglets could improve the gut microbiota composition [9,10,11,14] and promote the growth of recipient piglets [10,12,13]. Notably, indigenous breeds like NX pigs, characterized by distinct genetic backgrounds and metabolic traits, remain underexplored as FMT recipients. Here, we report that FMT from lean-type DLY pigs significantly increased the ADG in indigenous NX pigs, which is consistent with growth-promoting effects observed in prior FMT studies [13,14]. The nutrient digestion and utilization in pigs were closely linked to gut microbiota composition, which reflects the host’s growth, development, and overall health status [28]. In our study, lean DLY pig-derived fecal microbiota significantly enhanced the nutrient digestibility of Ca, P, CP, and CF in NX pigs. Given the documented differences in gut microbiota composition between fast-growing DLY and slow-growing NX pigs [16,29], we hypothesized that the phenotypic improvements observed in growth and digestion may be correlated with the microbial community changes induced by FMT. These were observations that suggest a potential link worthy of future mechanistic investigation. It was well known that serum biochemical parameters were commonly used to evaluate the physiological, metabolic, and immune status of animals. Among these, serum TP and GLB levels were closely associated with the growth performance of pigs [30]. Higher TP and GLB levels were indicative of efficient protein absorption utilization and a robust immune response [31]. In our study, FMT significantly increased the serum TP and GLB levels in NX pigs, which might have contributed to enhanced protein synthesis and metabolism, benefiting the health and weight gain of piglets. This finding was consistent with the observed improvements in CP digestibility and growth performance in NX pigs. Previous research has shown that transplantation of the fecal microbiota from adult pigs did not have affect the serum TP level of Landrace × Rongchang piglets [12], which could be attributable to some key elements, including FMT performed on pigs at different ages, in different breeds, and the composition of basal diets [13,32]. In summary, these results suggest that the improved growth performance in NX pigs post-FMT might be mediated by altered nutrient digestion and metabolism, and could be potentially driven by the intrinsic differences in gut microbiota composition between NX and DLY pigs.

While FMT has proven to be an effective method in modifying gut microbiota and improving phenotypes in recipient pigs, research has primarily focused on transfers to lean-type breeds from maternal or indigenous sources [33]. In contrast, the effect of transplantation of the fecal microbiota from lean-type pig breeds to indigenous pig breeds on the gut microbiota composition in recipient pigs remains limited. This study demonstrated that transplantation of the fecal microbiota from lean DLY pigs significantly restructured the gut microbiota of obese NX pigs, as evidenced by alterations in alpha and beta diversity, aligning with prior FMT findings [34]. Specifically, FMT enriched beneficial short-chain fatty acid (SCFA)-producing bacteria, including *Blautia*, *Faecalibacterium*, *Agathobacter*, *Olsenella*, and *Eubacterium_coprostanoligenes*_group, while suppressing *Lachnospiraceae*_XPB1014_group and *Treponema*. *Blautia*, a key SCFA producer within the Clostridium cluster XIVa—one of the two primary butyric acid-producing classes [35]—also generates succinate, lactate, and acetate as critical metabolic outputs, enhancing gut homeostasis and ameliorating metabolic disorders [36,37]. Critically, *Blautia* abundance exhibited a significant inverse association with key indicators of obesity and its obesity-related metabolic syndrome (e.g., serum lipids) [38,39]. In high-fat diet models, *Blautia* specifically improved serum lipid profile by decreasing TC, TG, and LDL-C [35]. This genus enrichment elevated fecal SCFA levels in mice models, activated the expression of hepatic LDL receptors and stimulated receptor-mediated uptake of LDL particles, thereby lowering LDL-C and promoting metabolic health [35,40]. In the present study, FMT intervention significantly enriched *Blautia* and reduced serum LDL-C levels in NX pigs, which was generally consistent with previous findings [35], although precise causal linkages warrant further investigation. *Blautia* also demonstrated significant potential as a modulator of nutrient utilization in swine. The enrichment of *Blautia* via FMT in NX pigs enhanced nutrient digestibility, particularly of CF and EE, and correlated positively with CP, EE, CF, Ca, and P utilization [41,42]. Mechanistically, *Blautia* harbored xylanolytic genes and encoded abundant carbohydrate-active enzymes (CAZymes) [42], which directly improved apparent ileal digestibility of key nutrients like threonine, tryptophan, CP, GE, and CF [43]. Its positive association with average daily feed intake in piglets might be linked to tryptophan metabolism [41], further driving improved ADG. Thus, *Blautia* enrichment likely enhanced NX pig growth performance via both CAZyme-mediated nutrient digestion metabolic regulation.

*Faecalibacterium*, a dominant genus in healthy gut microbiota, produced anti-inflammatory metabolites and played a crucial role in gut homeostasis and mucosal protection [44]. Its abundance was inversely associated with body mass index [45], highlighting its potential metabolic relevance. As a primary butyrate producer, *Faecalibacterium* growth positively correlates with butyrate synthesis, evidenced by isolates from healthy piglets metabolizing acetate to produce butyrate [46]. Interventional studies further demonstrate its therapeutic potential: FMT increased *Faecalibacterium* abundance [45], and *Faecalibacterium* supplementation attenuated hepatic inflammation, reduced lipid accumulation in high-fat diet fed mice [47,48]. Our study results were consistent with these findings, showing reduced LDL-C levels. We proposed this effect might involve *Faecalibacterium*’s anti-inflammatory actions, specifically the reduction in hepatic inflammatory cytokine levels (e.g., TNF-α, IL-8), which potentially upregulated the disheveled-binding antagonist of beta-catenin 3 and LDL receptor expression, enhancing hepatic LDL-C clearance [45,49]. Furthermore, *Faecalibacterium* has been a significantly positive correlate with enhanced growth performance, including higher ADG, in piglets [50,51]. Consistent with these reports, our study revealed significant enrichment of *Faecalibacterium* alongside slight positively associated with ADG in FMT-treated NX pigs. Another notable finding involves the inhibitory effect of *Faecalibacterium* on *Lachnospiraceae* colonization. As demonstrated in mice models, *Faecalibacterium* significantly reduced the abundance of *Lachnospiraceae*, and increased the abundance of CAZymes [52]. Critically, we observed a reduction in the abundance of the specific subgroup *Lachnospiraceae_XPB1014*_group. This subgroup exhibited negatively correlated with butyrate production, β-xylosidase activity (a key enzyme for hemicellulose degradation), and body weight [53,54], highlighted a novel antagonistic interaction in pigs. Our correlation analysis further revealed significant negative associations between *Lachnospiraceae*_XPB1014_group abundance and nutrient digestibility (CF, Ca, P), suggesting its suppression might contribute to enhanced nutrient utilization. Increased abundance of *Eubacterium_coprostanoligenes*_group and Escherichia-Shigella could also be linked to better feed efficiency, enhanced carbohydrate degradation, or higher acetate production in pigs [55,56]. Collectively, FMT reshaped the NX pig gut microbiota, especially by reducing the Lachnospiraceae_XPB1014_group while enriching butyrate producers (*Blautia*, *Eubacterium_coprostanoligenes*_group, *Faecalibacterium*). This shift likely improved ADG synergistically by enhancing nutrient digestibility through interconnected mechanisms involving specific microbial communities and enhancing systemic metabolic health post-FMT [57].

The serum metabolome, an intermediate phenotype bridging genomic information and final phenotypic expression, serves as a window into how gut microbiota influenced host metabolism [58]. In this study, FMT significantly altered serum metabolite profiles in NX pigs, revealing differential metabolites enriched in pathways related to the gut microbiome’s functional roles, such as lipid metabolism and amino acid metabolism. Levels of 5-hydroxy-L-tryptophan (a serotonin precursor), tyramine (synthesized from tyrosine), and aminoadipic acid (a lysine catabolite) decreased post-FMT. Elevated levels of 5-hydroxy-L-tryptophan and tyramin suppressed appetite [59], while reduced aminoadipic acid is associated with improved insulin sensitivity and inhibited inflammation [60,61]. Integrative analysis revealed significant positive correlations between *Lachnospiraceae*_XPB1014_group abundance and levels of 5-hydroxy-L-tryptophan, tyramine, and aminoadipic acid, while butyrate producer abundances were negatively correlated. This aligns with the evidence that *Faecalibacterium* supplementation significantly reduced aminoadipic acid and tyramine levels [52]. Collectively, the FMT-driven enrichment of beneficial butyrate producers, coupled with the decline in metabolites that have been linked to growth limitation, may contribute to the improved growth performance observed in NX pigs.

Emerging evidence underscored a significant role for the gut microbiota in regulating histidine metabolism. Supplementation with *Faecalibacterium* significantly reduced levels of histidinal biosynthetic precursors, urocanate, and imidazole-4-acetaldehyde [52]. Elevated histidine positively correlates with body mass index, and dietary histidine reduction might be a therapeutic strategy for obesity [62], partly because histidine suppresses food intake via hypothalamic conversion to histamine [63]. FMT increased *Faecalibacterium* abundance and decreased levels of histidinal (a direct histidine precursor) and its downstream metabolite formiminoglutamic acid. This suggests an association between Faecalibacterium enrichment and altered host histidine-related metabolism, which is consistent with earlier FMT studies reporting reduced plasma histidine in recipients receiving transplants from low-histidine donors [64]. However, whether this represents a direct microbial regulation of host biosynthesis requires further mechanistic investigation. Furthermore, the relative abundances of *Eubacterium_coprostanoligenes*_group and *Olsenella* were negatively correlated with 5-hydroxy-L-tryptophan, tyramine, aminoadipic acid, histidinal, and formiminoglutamic acid levels. In summary, the reduction in these growth-limiting metabolites (5-hydroxy-L-tryptophan, tyramine, aminoadipic acid, histidinal, formiminoglutamic acid) likely stemmed from the enrichment of *Faecalibacterium*, *Olsenella* and *Eubacterium_coprostanoligenes*_group, coupled with the reduction of the *Lachnospiraceae*_XPB1014_ group. This points to a microbiota-associated reprogramming of host amino acid metabolism, proposing a potential microbiota–metabolite axis that might support metabolic health and growth in NX pigs.

FMT also significantly altered serum metabolites involved in lipid metabolism. Sphingolipids produced by gut bacteria influence host metabolic processes. In the present study, FMT significantly increased the level of sphingolipid metabolites-3-dehydrosphinganine (a sphingosine precursor) level and significantly reduced the level of serum LDL-C in NX pigs. This improvement might involve sphingolipid metabolism. Crucially, we observed a strong positive correlation between 3-dehydrosphinganine levels and *Eubacterium_coprostanoligenes*_group abundance. This aligns with evidence that *Eubacterium_coprostanoligenes*_group influenced hyperlipidemia via sphingosine metabolism and the “glycosphingolipid biosynthesis” pathway [65]. It is thus speculated that the resulting sphingolipid intermediate metabolites possibly produced by this process may play a certain role in sphingolipid signaling crosstalk between gut microbiota and the host. Furthermore, *Faecalibacterium* supplementation significantly increased the sphingosine levels [52]. Sphingosine was phosphorylated by sphingosine kinase 1 to form sphingosine-1-phosphate (S1P), a bioactive lipid [66]. S1P enhanced LDL clearance via S1P3 receptor-mediated regulation of transendothelial LDL transport [67,68], and sphingosine supplementation itself reduces circulating TG, TC, and LDL-C [65]. We hypothesize that FMT-enriched *Eubacterium_coprostanoligenes*_group and *Faecalibacterium* might contribute to elevated levels of sphingosine-related precursors. Additionally, FMT also significantly reduced TXB2 levels, a marker of lipid peroxidation, which correlated negatively with *Faecalibacterium* abundance, suggesting this bacterium’s anti-inflammatory properties might indirectly mitigate lipid oxidation by inhibiting TXB2 synthesis [69]. Dodecanoic acid and its downstream metabolites exhibited antimicrobial properties, enhanced gut morphology, and improved growth performance in animals [70,71,72]. FMT significantly increased the level of dodecanoic acid, suggesting that this increase might have contributed to growth promotion in NX pigs. In summary, FMT improved the growth and metabolic health of NX pigs by reshaping gut microbiota composition and modifying serum metabolite profiles. This integrated analysis provided valuable insights into how targeted microbiota interventions could enhance nutrient metabolism in livestock, ultimately potentially improving growth performance. To establish mechanistic causality, however, gnotobiotic models or bacterial isolation studies are needed. Future studies should prioritize defined bacterial consortia (e.g., *Blautia*/*Faecalibacterium* blends) and host–microbe metabolic mapping. The findings of this study provide a basis for developing intervention strategies based on gut microbiota modulation. For instance, targeted probiotic preparations, prebiotic additives, or specific functional microbial consortia can be developed accordingly. By integrating these into diet formulations, intestinal health and metabolic status can be improved in finishing pigs under practical production conditions, thereby enhancing growth performance and feed utilization efficiency. This approach holds potential for application in the production systems of indigenous pig breeds, offering a feasible pathway to promote sustainable animal husbandry and advance precision nutrition technologies.

## 5. Conclusions

In conclusion, this study demonstrates that transplantation of the fecal microbiota from lean-type DLY pigs improved the growth of obese-type NX pigs by modulating the gut microbiota–host metabolic axis. Multi-omics analysis suggested that FMT reshaped the gut microbiome by enriching SCFA-producing bacteria (e.g., *Eubacterium*, *Blautia*, *Faecalibacterium*, and *Olsenella*) while reducing *Lachnospiraceae*_XPB1014_group. Critically, these microbial shifts drove functional reprogramming of host metabolism, including tryptophan, phenylalanine/tyrosine, histidine, and sphingolipid metabolism, thereby improving amino acid and lipid homeostasis. Through these mechanisms, FMT synergistically enhanced nutrient utilization, downregulated growth-limiting metabolites, modulated lipid homeostasis, and ultimately improved growth performance. These findings provide a novel strategy for optimizing the production of indigenous pig breeds through targeted gut microbiota modulation, filling a key gap in FMT applications for obese-type pigs, with significant implications for livestock farming.

## Figures and Tables

**Table 1:** Effects of transplantation of the fecal microbiota from DLY pigs on growth performance of NX pigs.

Items	CON ^1^	FMT ^2^	*p*-Value
Initial body weight, kg	19.37 ± 0.48	20.04 ± 0.49	0.336
Final body weight, kg	31.28 ± 1.07	33.83 ± 0.83	0.069
Average daily gain, kg/d	0.41 ± 0.02 ^a^	0.48 ± 0.01 ^b^	0.014
Average daily feed intake, kg/d	1.31 ± 0.01	1.34 ± 0.01	0.079
FCR, kg/kg	3.20 ± 0.10 ^b^	2.85 ± 0.07 ^a^	0.016

^1^ CON = control group (fed a basal diet + 10 mL/day normal saline); ^2^ FMT = FMT group (fed a basal diet + 10 mL/day of fecal microbiota suspension from age-matched DLY pig). Values are means ± SEM of individual measurements (*n* = 18). Values within the same row with different superscript letters (a, b) differ significantly (*p* < 0.05).

**Table 2:** Effects of transplantation of the fecal microbiota from DLY pigs on the coefficients of nutrient apparent digestibility of NX pigs.

Items	CON ^1^	FMT ^2^	*p*-Value
DM, %	87.60 ± 1.06	87.13 ± 0.60	0.690
EE, %	62.42 ± 0.89	66.57 ± 1.96	0.083
CP, %	91.74 ± 0.23	92.59 ± 0.26	0.053
CF, %	49.82 ± 0.80 ^a^	56.07 ± 2.02 ^b^	0.026
Ca, %	42.84 ± 0.70 ^a^	60.33 ± 0.21 ^b^	<0.01
P, %	37.48 ± 0.76 ^a^	47.70 ± 2.36 ^b^	<0.01

^1^ CON = control group (fed a basal diet + 10 mL/day normal saline); ^2^ FMT = FMT group (fed a basal diet + 10 mL/day of fecal microbiota suspension from age-matched DLY pig). Values are means ± SEM of individual measurements (*n* = 10). Values within the same row with different superscript letters (a, b) differ significantly (*p* < 0.05).

**Table 3:** Effects of transplantation of the fecal microbiota from DLY pigs on the serum biochemical indexes of NX pigs.

Items	CON ^1^	FMT ^2^	*p*-Value
LDL-C (mmol/L)	2.76 ± 0.15	1.72 ± 0.25	0.049
HDL-C (mmol/L)	1.32 ± 0.05	1.26 ± 0.08	0.451
TG (mmol/L)	0.51 ± 0.06	0.44 ± 0.12	0.605
TC (μmol/L)	2.96 ± 0.12	2.77 ± 0.21	0.419
CRE (μmol/L)	52.09 ± 3.35	51.7 ± 4.28	0.946
GLB (g/L)	52.8 ± 2.78 ^a^	60.67 ± 1.48 ^b^	0.027
ALB (g/L)	8.94 ± 1.14	9.9 ± 0.97	0.544
A/G	0.15 ± 0.02	0.16 ± 0.02	0.780
TP (g/L)	61.78 ± 1.92 ^a^	68.8 ± 0.71 ^b^	0.005
TBA (μmol/L)	9.85 ± 1.26	8.44 ± 2.6	0.592
ALT (U/L)	44.08 ± 1.91	48.1 ± 1.89	0.170

^1^ CON = control group (fed a basal diet + 10 mL/day normal saline); ^2^ FMT = FMT group (fed a basal diet + 10 mL/day of fecal microbiota suspension from age-matched DLY pig). Values are means ± SEM of individual measurements (*n* = 10). Values within the same row with different superscript letters (a, b) differ significantly (*p* < 0.05).

**Table 4:** Comparison of alpha diversity and richness indices of the fecal microbiota.

Items	CON ^1^	FMT ^2^	*p*-Value
Sobs	535.00 ± 17.61	444.00 ± 39.93	0.06
Ace	643.24 ± 20.46 ^b^	519.95 ± 47.00 ^a^	0.03
Chao1	665.03 ± 20.42 ^b^	526.01 ± 46.26 ^a^	0.02
Shannon	3.17 ± 0.15	3.33 ± 0.20	0.53
Simpson	0.15 ± 0.03	0.11 ± 0.02	0.31
Coverage	0.9994 ± 0.00	0.9995 ± 0.00	0.11

^1^ CON = control group (fed a basal diet + 10 mL/day normal saline); ^2^ FMT = FMT group (fed a basal diet + 10 mL/day of fecal microbiota suspension from age-matched DLY pig). Values are means ± SEM of individual measurements (*n* = 10). Values within the same row with different superscript letters (a, b) differ significantly (*p* < 0.05).

## Data Availability

Data is contained within the article and Supplementary Materials.

## Supplementary Materials

The following supporting information can be downloaded at https://www.mdpi.com/article/10.3390/ani16020177/s1, Table S1: Ingredient composition and nutrient content of basal diet. Figure S1: (A, B) OPLS-DA Permutation Test plots of serum metabolites in positive ion mode (A) and negative ion mode (B).

## Abbreviations

ADG, average daily weight gain; ALB, albumin; A/G, albumin–globulin ratio; ALT, alanine aminotransferase; BW, body weight; CF, crude fiber; CP, crude protein; CRE, serum creatinine; DLY, Duroc × (Landrace × Yorkshire); DM, dry matter; EE, ether extract; FMT, fecal microbiota transplantation; GLB, globulin; LEfSe, linear discriminant analysis effect size; NX, Ningxiang; OPLS-DA, Orthogonal partial least squares discriminant analysis; OTU, operational taxonomy unit; P, phosphorus; PCoA, principal co-ordinates analysis; SEM, standard error; TBA, total bile acid.

## References

[1] Zhu B., Gao H., Yang F., Li Y., Yang Q., Liao Y., Guo H., Xu K., Tang Z., Gao N. (2023). Comparative Characterization of Volatile Compounds of Ningxiang Pig, Duroc and Their Crosses (Duroc × Ningxiang) by Using SPME-GC-MS. Foods.

[2] Lei L., Wang Z., Li J., Yang H., Yin Y., Tan B., Chen J. (2021). Comparative Microbial Profiles of Colonic Digesta between Ningxiang Pig and Large White Pig. Animals.

[3] Lützhøft D.O., Bækgård C., Wimborne E., Straarup E.M., Pedersen K.M., Swann J.R., Pedersen H.D., Kristensen K., Morgills L., Nielsen D.S. (2024). High fat diet is associated with gut microbiota dysbiosis and decreased gut microbial derived metabolites related to metabolic health in young Göttingen Minipigs. PLoS ONE.

[4] Gong Y., Zou X., Xia W., Wen X., Zhang X., Xiao Y., Yang H. (2019). Comparative metabolomic analysis of caecal digesta between Jinhua pig and Landrace pig. Czech J. Anim. Sci..

[5] Yang L., Liu X., Huang X., Zhang L., Yan H., Hou X., Wang L., Wang L. (2023). Metabolite and Proteomic Profiling of Serum Reveals the Differences in Molecular Immunity between Min and Large White Pig Breeds. Int. J. Mol. Sci..

[6] Zhang C., Lu W., Liu H., Shen L., Zhu M., Zhou T., Zhang L., Xiao D., Chen L. (2024). Rumen Microbiota Transplantation Alleviates Gossypol Diet-Induced Reproductive, Liver, Intestinal Damage in Male Mice. Animals.

[7] Diao H., Xiao Y., Yan H.L., Yu B., He J., Zheng P., Yu J., Mao X.B., Chen D.W. (2021). Effects of Early Transplantation of the Faecal Microbiota from Tibetan Pigs on the Gut Development of DSS-Challenged Piglets. BioMed Res. Int..

[8] Zhou H., Sun J., Yu B., Liu Z., Chen H., He J., Mao X., Zheng P., Yu J., Luo J. (2021). Gut microbiota absence and transplantation affect growth and intestinal functions: An investigation in a germ-free pig model. Anim. Nutr..

[9] Tang W., Chen D., Yu B., He J., Huang Z., Zheng P., Mao X., Luo Y., Luo J., Wang Q. (2020). Capsulized faecal microbiota transplantation ameliorates post-weaning diarrhoea by modulating the gut microbiota in piglets. Vet. Res..

[10] Cheng C.S., Wei H.K., Wang P., Yu H.C., Zhang X.M., Jiang S.W., Peng J. (2019). Early intervention with faecal microbiota transplantation: An effective means to improve growth performance and the intestinal development of suckling piglets. Animal.

[11] Teng T., Gao F., He W., Fu H., Guo J., Bai G., Shi B. (2020). An Early Fecal Microbiota Transfer Improves the Intestinal Conditions on Microflora and Immunoglobulin and Antimicrobial Peptides in Piglets. J. Agric. Food Chem..

[12] Qi R., Zhang Z., Wang J., Qiu X., Wang Q., Yang F., Huang J., Liu Z. (2021). Introduction of Colonic and Fecal Microbiota from an Adult Pig Differently Affects the Growth, Gut Health, Intestinal Microbiota and Blood Metabolome of Newborn Piglets. Front. Microbiol..

[13] Hu L., Geng S., Li Y., Cheng S., Fu X., Yue X., Han X. (2017). Exogenous Fecal Microbiota Transplantation from Local Adult Pigs to Crossbred Newborn Piglets. Front. Microbiol..

[14] Li H., Han L., Zhou F., Wu Z., Zhang L., Xie R., Jiang F., Tian Q., Huang X. (2024). Ningxiang Pig-Derived Microbiota Affects the Growth Performance, Gut Microbiota, and Serum Metabolome of Nursery Pigs. Animals.

[15] Diao H., Yan H.L., Xiao Y., Yu B., Zheng P., He J., Yu J., Mao X.B., Chen D.W. (2018). Modulation of intestine development by fecal microbiota transplantation in suckling pigs. RSC Adv..

[16] Yin J., Li Y., Tian Y., Zhou F., Ma J., Xia S., Yang T., Ma L., Zeng Q., Liu G. (2023). Obese Ningxiang pig-derived microbiota rewires carnitine metabolism to promote muscle fatty acid deposition in lean DLY pigs. Innovation.

[17] Yang T., Liu Y., Yin J., Yv T., Zhou F., Li Y., Yang L., Han L., Huang X. (2024). Transplantation of fecal microbiota from different breeds improved intestinal barrier condition and modulated ileal microflora of recipient pigs. J. Anim. Sci..

[18] Hu J., Chen L., Tang Y., Xie C., Xu B., Shi M., Zheng W., Zhou S., Wang X., Liu L. (2018). Standardized Preparation for Fecal Microbiota Transplantation in Pigs. Front. Microbiol..

[19] Meurens F., Summerfield A., Nauwynck H., Saif L., Gerdts V. (2012). The pig: A model for human infectious diseases. Trends Microbiol..

[20] Zhang C., Liu B., Cui Z., Wu K., Huang H., Wang Y., Ma X., Tan B. (2025). Effects of Magnolia officinalis extract on the growth performance and immune function of weaned piglets. Porc. Health Manag..

[21] Wu J.P., Zhou R., Liu L.S., Casper D.P., Lang X., Wang C.L., Zhang L.P., Wei S., Liu H.B. (2021). Growth performance, nutrient digestibility, blood parameters, and carcass characteristics by lambs fed an oregano and cobalt blend. Animal.

[22] He J., Li X., Yan M., Chen X., Sun C., Tan J., Song Y., Xu H., Wu L., Yang Z. (2024). Inulin Reduces Kidney Damage in Type 2 Diabetic Mice by Decreasing Inflammation and Serum Metabolomics. J. Diabetes Res..

[23] Guo P., Zeng M., Zhang Y., Zhang Z., Wu Y., Ye K., Chang F., Wang Y., Zheng X., Feng W. (2024). Integration strategies involving 16S rDNA sequencing combined with untargeted metabolomics revealed the mechanism of *Selaginella tamariscina* (Beauv.) Spring in db/db diabetic mice. Biomed. Pharmacother..

[24] Hu J., Chen J., Xu X., Hou Q., Ren J., Yan X. (2023). Gut microbiota-derived 3-phenylpropionic acid promotes intestinal epithelial barrier function via AhR signaling. Microbiome.

[25] Ma J., Duan Y., Li R., Liang X., Li T., Huang X., Yin Y., Yin J. (2022). Gut microbial profiles and the role in lipid metabolism in Shaziling pigs. Anim. Nutr..

[26] Jiang W., Zhang Y., Cheng H., Hu X., You W., Song E., Hu Z., Jiang F. (2024). Fermented Palm Kernel Cake Improves the Rumen Microbiota and Metabolome of Beef Cattle. Animals.

[27] Zhang W., Zou G., Li B., Du X., Sun Z., Sun Y., Jiang X. (2020). Fecal Microbiota Transplantation (FMT) Alleviates Experimental Colitis in Mice by Gut Microbiota Regulation. J. Microbiol. Biotechnol..

[28] Muramatsu M.K., Winter S.E. (2024). Nutrient acquisition strategies by gut microbes. Cell Host Microbe.

[29] Xing Y., Wu X., Xie C., Xiao D., Zhang B. (2020). Meat Quality and Fatty Acid Profiles of Chinese Ningxiang Pigs Following Supplementation with N-Carbamylglutamate. Animals.

[30] Liu Y., Huo B., Chen Z., Wang K., Huang L., Che L., Feng B., Lin Y., Xu S., Zhuo Y. (2023). Effects of Organic Chromium Yeast on Performance, Meat Quality, and Serum Parameters of Grow-Finish Pigs. Biol. Trace Elem. Res..

[31] Xu S., Shi J., Shi X., Dong Y., Wu X., Li Z., Fang Z., Lin Y., Che L., Li J. (2018). Effects of dietary supplementation with lysozyme during late gestation and lactation stage on the performance of sows and their offspring. J. Anim. Sci..

[32] Wang X., Tsai T., Deng F., Wei X., Chai J., Knapp J., Apple J., Maxwell C.V., Lee J.A., Li Y. (2019). Longitudinal investigation of the swine gut microbiome from birth to market reveals stage and growth performance associated bacteria. Microbiome.

[33] Canibe N., O’Dea M., Abraham S. (2019). Potential relevance of pig gut content transplantation for production and research. J. Anim. Sci. Biotechnol..

[34] McCormack U.M., Curião T., Wilkinson T., Metzler-Zebeli B.U., Reyer H., Ryan T., Calderon-Diaz J.A., Crispie F., Cotter P.D., Creevey C.J. (2018). Fecal Microbiota Transplantation in Gestating Sows and Neonatal Offspring Alters Lifetime Intestinal Microbiota and Growth in Offspring. mSystems.

[35] Yang Y.N., Wang Q.C., Xu W., Yu J., Zhang H., Wu C. (2022). The berberine-enriched gut commensal Blautia producta ameliorates high-fat diet (HFD)-induced hyperlipidemia and stimulates liver LDLR expression. Biomed. Pharmacother..

[36] Hosomi K., Saito M., Park J., Murakami H., Shibata N., Ando M., Nagatake T., Konishi K., Ohno H., Tanisawa K. (2022). Oral administration of Blautia wexlerae ameliorates obesity and type 2 diabetes via metabolic remodeling of the gut microbiota. Nat. Commun..

[37] Liu X., Mao B., Gu J., Wu J., Cui S., Wang G., Zhao J., Zhang H., Chen W. (2021). Blautia—A new functional genus with potential probiotic properties?. Gut Microbes.

[38] Wu X.R., Chen Z.Z., Dong X.L., Zhao Q.P., Cai J. (2023). A Novel Symbiotic Formulation Reduces Obesity and Concomitant Metabolic Syndrome in Rats by Raising the Relative Abundance of Blautia. Nutrients.

[39] Benítez-Páez A., Gómez Del Pugar E.M., López-Almela I., Moya-Pérez Á., Codoñer-Franch P., Sanz Y. (2020). Depletion of Blautia Species in the Microbiota of Obese Children Relates to Intestinal Inflammation and Metabolic Phenotype Worsening. mSystems.

[40] Zhao Y., Liu J., Hao W., Zhu H., Liang N., He Z., Ma K.Y., Chen Z.Y. (2017). Structure-Specific Effects of Short-Chain Fatty Acids on Plasma Cholesterol Concentration in Male Syrian Hamsters. J. Agric. Food Chem..

[41] Zhu J.J., Gao M.X., Song X.J., Zhao L., Li Y.W., Hao Z.H. (2018). Changes in bacterial diversity and composition in the faeces and colon of weaned piglets after feeding fermented soybean meal. J. Med. Microbiol..

[42] Singh R.P., Bhaiyya R., Thakur R., Niharika J., Singh C., Latousakis D., Saalbach G., Nepogodiev S.A., Singh P., Sharma S.C. (2022). Biochemical Basis of Xylooligosaccharide Utilisation by Gut Bacteria. Int. J. Mol. Sci..

[43] Hong J., Halbur J., Petry A.L., Doung T., Llamas-Moya S., Kitt S., Bertram M., Weaver E. (2025). Effects of a fiber-degrading enzyme on ileal digestibility of amino acids and fiber and total tract digestibility of energy and fiber in growing pigs fed diets with high level of corn distiller grains with solubles. J. Anim. Sci..

[44] Sokol H., Seksik P., Furet J.P., Firmesse O., Nion-Larmurier I., Beaugerie L., Cosnes J., Corthier G., Marteau P., Doré J. (2009). Low counts of *Faecalibacterium prausnitzii* in colitis microbiota. Inflamm. Bowel Dis..

[45] Martín R., Rios-Covian D., Huillet E., Auger S., Khazaal S., Bermúdez-Humarán L.G., Sokol H., Chatel J.M., Langella P. (2023). Faecalibacterium: A bacterial genus with promising human health applications. FEMS Microbiol. Rev..

[46] Foditsch C., Santos T.M., Teixeira A.G., Pereira R.V., Dias J.M., Gaeta N., Bicalho R.C. (2014). Isolation and characterization of *Faecalibacterium prausnitzii* from calves and piglets. PLoS ONE.

[47] Yang M., Wang J.H., Shin J.H., Lee D., Lee S.N., Seo J.G., Shin J.-H., Nam Y.-D., Kim H., Sun X. (2023). Pharmaceutical efficacy of novel human-origin *Faecalibacterium prausnitzii* strains on high-fat-diet-induced obesity and associated metabolic disorders in mice. Front. Endocrinol..

[48] Munukka E., Rintala A., Toivonen R., Nylund M., Yang B., Takanen A., Hänninen A., Vuopio J., Huovinen P., Jalkanen S. (2017). *Faecalibacterium prausnitzii* treatment improves hepatic health and reduces adipose tissue inflammation in high-fat fed mice. ISME J..

[49] Lenoir M., Martín R., Torres-Maravilla E., Chadi S., González-Dávila P., Sokol H., Langella P., Chain F., Bermúdez-Humarán L.G. (2020). Butyrate mediates anti-inflammatory effects of *Faecalibacterium prausnitzii* in intestinal epithelial cells through Dact3. Gut Microbes.

[50] Mahmud M.R., Jian C., Uddin M.K., Huhtinen M., Salonen A., Peltoniemi O., Venhoranta H., Oliviero C. (2023). Impact of Intestinal Microbiota on Growth Performance of Suckling and Weaned Piglets. Microbiol. Spectr..

[51] Gaukroger C.H., Stewart C.J., Edwards S.A., Walshaw J., Adams I.P., Kyriazakis I. (2020). Changes in Faecal Microbiota Profiles Associated with Performance and Birthweight of Piglets. Front. Microbiol..

[52] Geng P., Zhao N., Zhou Y., Harris R.S., Ge Y. (2025). *Faecalibacterium prausnitzii* regulates carbohydrate metabolic functions of the gut microbiome in C57BL/6 mice. Gut Microbes.

[53] Bai Y., Zhou X., Li N., Zhao J., Ye H., Zhang S., Yang H., Pi Y., Tao S., Han D. (2021). In Vitro Fermentation Characteristics and Fiber-Degrading Enzyme Kinetics of Cellulose, Arabinoxylan, β-Glucan and Glucomannan by Pig Fecal Microbiota. Microorganisms.

[54] Hu C., Li F., Duan Y., Yin Y., Kong X. (2019). Glutamic acid supplementation reduces body fat weight in finishing pigs when provided solely or in combination with arginine and it is associated with colonic propionate and butyrate concentrations. Food Funct..

[55] Niu Q., Pu G., Fan L., Gao C., Lan T., Liu C., Du T., Kim S.W., Niu P., Zhang Z. (2022). Identification of Gut Microbiota Affecting Fiber Digestibility in Pigs. Curr. Issues Mol. Biol..

[56] McCormack U.M., Curião T., Metzler-Zebeli B.U., Wilkinson T., Reyer H., Crispie F., Cotter P.D., Creevey C.J., Gardiner G.E., Lawlor P.G. (2019). Improvement of Feed Efficiency in Pigs through Microbial Modulation via Fecal Microbiota Transplantation in Sows and Dietary Supplementation of Inulin in Offspring. Appl. Environ. Microbiol..

[57] Visuthranukul C., Sriswasdi S., Tepaamorndech S., Chamni S., Leelahavanichkul A., Joyjinda Y., Aksornkitti V., Chomtho S. (2024). Enhancing gut microbiota and microbial function with inulin supplementation in children with obesity. Int. J. Obes..

[58] Liu G., Yu Q., Tan B., Ke X., Zhang C., Li H., Zhang T., Lu Y. (2022). Gut dysbiosis impairs hippocampal plasticity and behaviors by remodeling serum metabolome. Gut Microbes.

[59] Van Galen K.A., Horst K.W.T., Serlie M.J. (2021). Serotonin, food intake, obesity. Obes. Rev..

[60] Chang A.Y., Asokan A.K., Lalia A.Z., Sakrikar D., Lanza I.R., Petterson X.M., Nair K.S. (2024). Insulin Regulation of Lysine and α-Aminoadipic Acid Dynamics and Amino Metabolites in Women with and Without Insulin Resistance. Diabetes.

[61] Wang T., Wu H., Shi X., Dai M., Liu Y. (2024). Aminoadipic acid aggravates atherosclerotic vascular inflammation through ROS/TXNIP/NLRP3 pathway, a harmful microbial metabolite reduced by paeonol. Int. J. Biochem. Cell Biol..

[62] Flores V., Spicer A.B., Sonsalla M.M., Richardson N.E., Yu D., Sheridan G.E., Trautman M.E., Babygirija R., Cheng E.P., Rojas J.M. (2023). Regulation of metabolic health by dietary histidine in mice. J. Physiol..

[63] Moro J., Tomé D., Schmidely P., Demersay T.C., Azzout-Marniche D. (2020). Histidine: A Systematic Review on Metabolism and Physiological Effects in Human and Different Animal Species. Nutrients.

[64] Quesada-Vázquez S., Castells-Nobau A., Latorre J., Oliveras-Cañellas N., Puig-Parnau I., Tejera N., Tobajas Y., Baudin J., Hildebrand F., Beraza N. (2023). Potential therapeutic implications of histidine catabolism by the gut microbiota in NAFLD patients with morbid obesity. Cell Rep. Med..

[65] Wei W., Jiang W., Tian Z., Wu H., Ning H., Yan G., Zhang Z., Li Z., Dong F., Sun Y. (2021). Fecal g. Streptococcus and g. Eubacterium_coprostanoligenes_group combined with sphingosine to modulate the serum dyslipidemia in high-fat diet mice. Clin. Nutr..

[66] Kwong E., Li Y., Hylemon P.B., Zhou H. (2015). Bile acids and sphingosine-1-phosphate receptor 2 in hepatic lipid metabolism. Acta Pharm. Sin. B.

[67] Velagapudi S., Wang D., Poti F., Feuerborn R., Robert J., Schlumpf E., Yalcinkaya M., Panteloglou G., Potapenko A., Simoni M. (2024). Sphingosine-1-phosphate receptor 3 regulates the transendothelial transport of high-density lipoproteins and low-density lipoproteins in opposite ways. Cardiovasc. Res..

[68] Jang E., Robert J., Rohrer L., von Eckardstein A., Lee W.L. (2020). Transendothelial transport of lipoproteins. Atherosclerosis.

[69] Wang T., Chen X., Li H., Chen W., Xu Y., Yao Y., Zhang H., Han Y., Zhang L., Que C. (2022). Pro-thrombotic changes associated with exposure to ambient ultrafine particles in patients with chronic obstructive pulmonary disease: Roles of lipid peroxidation and systemic inflammation. Part. Fibre Toxicol..

[70] Ficagna C.A., Galli G.M., Zatti E., Zago I., Amaral M., de Vitt M.G., Paiano D., da Silva A.S. (2024). Addition of Butyric Acid and Lauric Acid Glycerides in Nursery Pig Feed to Replace Conventional Growth Promoters. Animals.

[71] Zeng X., Yang Y., Wang J., Wang Z., Li J., Yin Y., Yang H. (2022). Dietary butyrate, lauric acid and stearic acid improve gut morphology and epithelial cell turnover in weaned piglets. Anim. Nutr..

[72] Wu Y., Zhang H., Zhang R., Cao G., Li Q., Zhang B., Wang Y., Yang C. (2021). Serum metabolome and gut microbiome alterations in broiler chickens supplemented with lauric acid. Poult. Sci..

