# Supplementary material for: Lean DLY Pig-Derived Fecal Microbiota Promotes Growth Performance by Modulating Gut Microbiota: Serum Metabolic Profiles in Obese Ningxiang Pigs
Source: Animals (Basel). 2026 Jan 7;16(2):177. doi: 10.3390/ani16020177 (PMC12838150; doi:10.3390/ani16020177)
Supplement: Supplementary file 1 [file animals-16-00177-s001.zip › animals-4029757-supplementary.pdf]

Table S1. Compositions and nutrient levels of the basal diet (air-dry basis)

| Ingredients | Contents (%) |
|---|---|
| Corn | 57.50 |
| Full-fat rice bran | 15.00 |
| Wheat bran | 10.00 |
| Soybean meal 43% | 14.00 |
| Stone powder | 1.20 |
| DL-methionine | 0.05 |
| L-lysine | 0.64 |
| L-threonine | 0.15 |
| Calcium hydrogen phosphate | 0.50 |
| Choline chloride 50% | 0.10 |
| Salt | 0.36 |
| Premix1 | 0.50 |
| Total | 100.00 |
| Nutrient levels2 | |
| Dry matter | 86.64 |
| Crude protein | 14.64 |
| Crude fiber | 3.94 |
| Crude fat | 5.11 |
| Total calcium | 0.66 |
| Total phosphorus | 0.65 |
| Gross energy, GE (MJ/kg) | 16.22 |
| Digestible energy, (MJ/kg) | 13.19 |
| Lysine | 0.88 |
| Methionine | 0.82 |
| Cysteine | 0.61 |
| Tryptophan | 0.81 |

[1]The premix provided the following per kg of the diet: D-Calcium Pantothenate 21.00 mg, Zn 102.38 mg, Cu 108.75 mg, Mn 59.63 mg, Fe 180.00 mg, Se 0.53 mg, I 1.05 mg, VA 9,750.00 IU, VB1 3.00 mg, VB2 7.50 mg, VB6 4.50 mg, VB12 30.00 μg, VD3 3,000.00 IU, VK3 3.00 mg, VE 24.00 mg, nicotinamide 36.00 mg, biotin 0.15 mg, folic acid 1.50 mg, titanium dioxide 2.5 g.

[2] Nutrient levels were calculated using NRC values (2012), standardized ileal digestibility coefficients, and ingredient matrix values.[EE1]

A

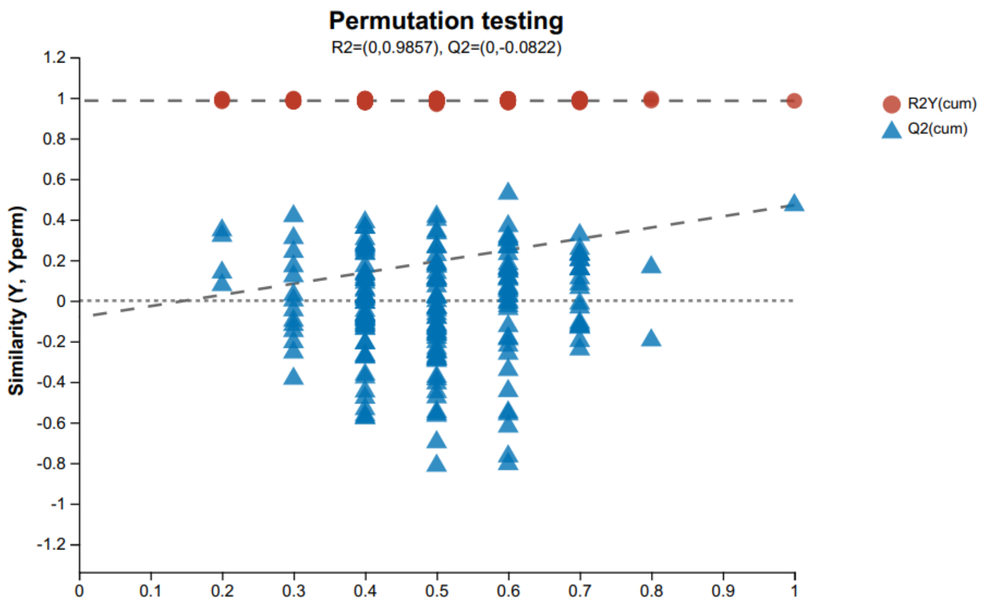

B

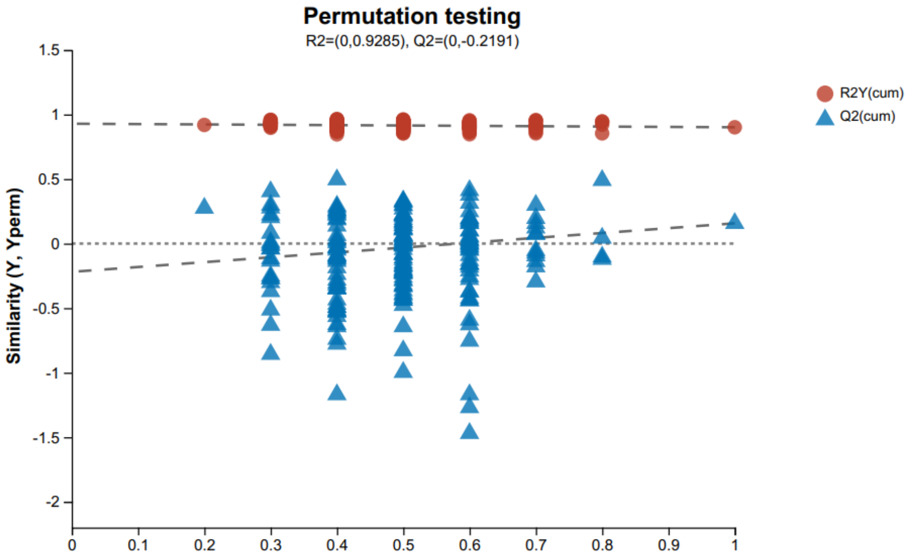


Figure S1. (A, B) OPLS-DA Permutation Test plots of serum metabolites in positive ion mode (A) and negative ion mode (B). Data represent the means ± SEM (n=10).
